# Expansion of the measles and rubella laboratory network, India

**DOI:** 10.2471/BLT.21.286999

**Published:** 2022-02-03

**Authors:** Deepa Sharma, Lucky Sangal, Neetu Vijay, Uma Nalavade, Kaveri Krishnasamy, Shailesh Pawar, Harmanmeet Kaur, Jitendra Narayan, Sneha Rane, Manish Narkar, Ramesh Arumugam, Dhanagaran D, AP Sugunan, Anukumar Balakrishnan, Bestin Joseph, Jyotirmayee Turuk, Jyotsnamayee Sabat, Prakash Sahoo, Pradip Barde, Lalit Sahare, Mahendra Ukey, Manoj Kumar, Nikesh Sinha, Zulfiquar Ali Bhuttoo, Paluru Vijayachari, Punnam Chander, Shivangi Sharma, Venkatesha D, Gayathree L, Chethan Sharma, Pankaj Bhatnagar, Kristin VanderEnde, Nirmal Kaundal, Ratnesh Murugan, Pradeep Haldar, Deepak Gadkari, Neeraj Aggarwal, Nivedita Gupta

**Affiliations:** a Indian Council of Medical Research (ICMR)–National Institute of Virology, Mumbai Unit, Mumbai, India.; b Regional Office for South-East Asia, World Health Organization, New Delhi, India.; c Virology Unit, Division of Epidemiology & Communicable Diseases, Indian Council of Medical Research, V. Ramalingaswami Bhawan, P.O. Box No. 4911 Ansari Nagar, New Delhi - 110029, India.; d King Institute of Preventive Medicine, Guindy, Chennai, India.; e ICMR–National Institute of Virology, Field Unit, Alappuzha, Kerala, India.; f ICMR–Regional Medical Research Centre, Bhubaneswar, India.; g ICMR–National Institute for Research in Tribal Health, Jabalpur, Madhya Pradesh, India.; h Rajendra Institute of Medical Science, Ranchi, Jharkhand, India.; i ICMR–Regional Medical Research Centre, Port Blair, Andaman and Nicobar Islands, India.; j Hassan Institute of Medical Sciences, Hassan, Karnataka, India.; k National Public Health Support Programme, World Health Organization, New Delhi, India.; l National Health Mission, Ministry of Health and Family Welfare, New Delhi, India.; m Indian Council of Medical Research, New Delhi, India.

## Abstract

**Objective:**

To expand the measles and rubella laboratory network of India by integrating new laboratories.

**Methods:**

In collaboration with the World Health Organization (WHO), the Indian government developed a 10-step scheme to systematically expand the number of laboratories performing serological and molecular testing for measles and rubella. The Indian Council of Medical Research and WHO identified suitable laboratories based on their geographical location, willingness, preparedness, past performance and adherence to national quality control and quality assurance mechanisms. The 10-step scheme was initiated with training on measles and rubella diagnostic assays followed by testing of both measles and rubella serology and molecular unknown panels, cross-verification with reference laboratories and ended with WHO on-site accreditation.

**Findings:**

After extensive training, technical support, funding and monitoring, all six selected laboratories attained passing scores of 90.0% or more in serological and molecular proficiency testing of measles and rubella. Since 2018, the laboratories are a part of the measles and rubella network of India. Within 12 months of initiation of independent reporting, the six laboratories have tested 2287 serum samples and 701 throat or nasopharyngeal swabs or urine samples.

**Conclusion:**

The process led to strengthening and expansion of the network. This proficient laboratory network has helped India in scaling up serological and molecular testing of measles and rubella while ensuring high quality testing. The collaborative model developed by the Indian government with WHO can be implemented by other countries for expanding laboratory networks for surveillance of measles and rubella as well as other infectious diseases.

## Résumé

**Objectif:**

Étendre le réseau de laboratoires d'analyse de la rougeole et de la rubéole en Inde en y intégrant de nouveaux établissements.

**Méthodes:**

En collaboration avec l'Organisation mondiale de la Santé (OMS), le gouvernement indien a mis au point un programme en 10 étapes destiné à accroître systématiquement le nombre de laboratoires effectuant des tests sérologiques et moléculaires de détection de la rougeole et de la rubéole. Le Conseil indien de la recherche médicale et l'OMS ont identifié une série de laboratoires éligibles en raison de leur situation géographique, de leur volonté d'y participer, de leur niveau de préparation, de leurs performances antérieures et de leur respect des mécanismes nationaux de contrôle et d'assurance qualité. Ce programme en 10 étapes a commencé par une formation aux analyses diagnostiques pour la rougeole et la rubéole, suivie de la réalisation de tests sérologiques et moléculaires pour détecter la rougeole et la rubéole sur des échantillons inconnus, une vérification croisée avec les laboratoires de référence et enfin, une accréditation OMS sur site.

**Résultats:**

Après avoir reçu une formation complète, un soutien technique, un financement et fait l'objet d'une surveillance, les six laboratoires sélectionnés ont tous réussi, obtenant une note égale ou supérieure à 90,0% dans les tests d'aptitude au dépistage sérologique et moléculaire de la rougeole et de la rubéole. Depuis 2018, ces laboratoires font partie du réseau d'analyse de la rougeole et de la rubéole en Inde. Douze mois après l'établissement des premiers rapports indépendants, les six laboratoires avaient testé 2287 échantillons de sérum et 701 écouvillons nasopharyngés, prélèvements de gorge ou échantillons d'urine.

**Conclusion:**

Le programme a permis de renforcer et d'étendre le réseau de laboratoires compétents. L'Inde a ainsi pu développer un système de tests sérologiques et moléculaires de haute qualité pour dépister la rougeole et la rubéole. Le modèle collaboratif adopté par le gouvernement indien avec l'OMS pourrait s'appliquer à d'autres pays qui souhaitent étendre leur réseau de laboratoires chargés de surveiller la rougeole et la rubéole, mais aussi d'autres maladies infectieuses.

## Resumen

**Objetivo:**

Ampliar la red de laboratorios para el diagnóstico del sarampión y la rubéola en la India mediante el establecimiento de nuevos laboratorios.

**Métodos:**

En colaboración con la Organización Mundial de la Salud (OMS), el gobierno de la India desarrolló un programa de 10 pasos para ampliar de manera sistemática el número de laboratorios que realizan pruebas serológicas y moleculares para detectar el sarampión y la rubéola. El Consejo Indio de Investigación Médica y la OMS identificaron los laboratorios adecuados según su ubicación geográfica, su disposición, su preparación, sus resultados anteriores y su adhesión a los mecanismos nacionales de control y garantía de calidad. El programa de 10 pasos se inició con la formación sobre las pruebas de diagnóstico del sarampión y la rubéola, seguida de la realización de pruebas serológicas y moleculares para el sarampión y la rubéola en grupos desconocidos, la verificación cruzada con los laboratorios de referencia y finalizó con la acreditación *in situ* a cargo de la OMS.

**Resultados:**

Luego de una extensa formación, apoyo técnico, financiamiento y seguimiento, los seis laboratorios seleccionados alcanzaron puntajes de aprobación del 90,0 % o más en las pruebas de aptitud serológica y molecular para el sarampión y la rubéola. Desde 2018, los laboratorios forman parte de la red contra el sarampión y la rubéola de la India. En los 12 meses posteriores al inicio de la presentación de informes independientes, los seis laboratorios han analizado 2287 muestras de suero y 701 exudados faríngeos o nasofaríngeos, o muestras de orina.

**Conclusión:**

El proceso condujo al fortalecimiento y la expansión de la red. Esta competente red de laboratorios ha ayudado a India a ampliar las pruebas serológicas y moleculares para el sarampión y la rubéola, garantizando al mismo tiempo la alta calidad de las pruebas. El modelo de colaboración que ha desarrollado el gobierno de la India con la OMS se puede aplicar en otros países para ampliar las redes de laboratorios con el fin de vigilar los casos de sarampión y rubéola, así como otras enfermedades infecciosas.

## ملخص

**الغرض:**

توسيع شبكة مختبرات الحصبة والحصبة الألمانية في الهند من خلال دمج مختبرات جديدة.

**الطريقة:**

بالتعاون مع منظمة الصحة العالمية (WHO)، قامت الحكومة الهندية بتطوير مخطط يتألف من 10 خطوات بهدف زيادة عدد المختبرات التي تقوم بإجراء الاختبارات المصلية والجزيئية للحصبة والحصبة الألمانية، بشكل منهجي. حدد كل من المجلس الهندي للبحوث الطبية ومنظمة الصحة العالمية المختبرات المناسبة بناءً على موقعها الجغرافي، ورغبتها، واستعدادها، وأدائها السابق، والتزامها بالآليات الوطنية لمراقبة الجودة وتوكيد الجودة. تم بدء مخطط الخطوات العشر بالتدريب على الفحوصات التشخيصية للحصبة والحصبة الألمانية، وتبعها اختبار كل من مصل الحصبة والحصبة الألمانية والقوائم الجزيئية غير المعروفة، والتحقق المتبادل مع المختبرات المرجعية، وانتهى بالاعتماد الميداني لدى منظمة الصحة العالمية.

**النتائج:**

بعد التدريب المكثف، والدعم الفني، والتمويل، والمراقبة، حققت كل المختبرات الستة المختارة على درجات اجتياز بلغت 90.0% أو أكثر، في اختبار الكفاءة المصلية والجزيئية للحصبة والحصبة الألمانية. منذ عام 2018، أصبحت المختبرات جزءًا من شبكة الحصبة والحصبة الألمانية في الهند. في غضون 12 شهرًا من بدء تقديم التقارير المستقلة، قامت المختبرات الستة باختبار 2287 عينة مصل و701 عينة من الحلق، أو البلعوم الأنفي، أو عينة بول.

**الاستنتاج:**

أدت تلك العملية إلى تعزيز وتوسيع الشبكة. ساعدت هذه الشبكة المتمكنة من المختبرات، الهند في توسيع نطاق الاختبارات المصلية والجزيئية للحصبة والحصبة الألمانية، مع ضمان إجراء اختبارات عالية الجودة. يمكن تنفيذ النموذج التعاوني الذي طورته الحكومة الهندية مع منظمة الصحة العالمية بواسطة دول أخرى لتوسيع شبكات المختبرات، من أجل مراقبة الحصبة والحصبة الألمانية فضلاً عن الأمراض المعدية الأخرى.

## 摘要

**目的:**

旨在通过整合新的实验室，来扩大印度的麻疹和风疹实验室网络。

**方法:**

印度政府与世界卫生组织 (WHO) 合作制定了一个 10 步计划，以系统地扩大对麻疹和风疹进行血清学和分子检测的实验室的数量。印度医学研究委员会和世界卫生组织根据其地理位置、意愿、准备情况、过去的表现以及对国家质量控制和质量保证机制的遵守情况，确定了适当的实验室。该 10 步计划从麻疹和风疹诊断检测培训开始，随后测试麻疹和风疹血清学和未知分子小组，与参考实验室进行交叉验证，最后获得世卫组织现场认证。

**结果:**

经过广泛的培训、技术支持、资金支持和监测，所有六个选定的实验室在麻疹和风疹的血清学和分子水平测试中均获得了 90.0% 或更高的通过分数。自 2018 年以来，这些实验室成为印度麻疹和风疹网络的一部分。在独立报告开始后的 12 个月内，六个实验室已检测了 2287 份血清样本和 701 份咽喉或鼻咽拭子或尿液样本。

**结论:**

这个过程使得网络得到加强和扩展。这个熟练的实验室网络帮助印度扩大了麻疹和风疹的血清学和分子检测，同时确保了高质量的检测。印度政府与世卫组织开发的合作模式可以在其他国家实施，以扩大监测麻疹和风疹以及其他传染病的实验室网络。

## Резюме

**Цель:**

Расширить сеть лабораторий для исследования кори и краснухи в Индии путем интеграции новых лабораторий.

**Методы:**

Правительство Индии в сотрудничестве со Всемирной организацией здравоохранения (ВОЗ) разработало 10-этапную схему систематического увеличения числа лабораторий, проводящих серологические и молекулярные тесты на корь и краснуху. Индийский совет по медицинским исследованиям и ВОЗ определили подходящие для этой цели лаборатории с учетом их географического положения, стремления участвовать, подготовленности, опыта работы и соблюдения национальных механизмов контроля и обеспечения качества. 10-этапная схема началась с обучения по проведению диагностических тестов на корь и краснуху, затем серологических тестов на корь и краснуху, а также с обучения использованию панелей молекулярной диагностики, продолжилась проведением перекрестной проверки с референс-лабораториями и завершилась аккредитацией ВОЗ на местах.

**Результаты:**

После обширного обучения, технической поддержки, финансирования и мониторинга все шесть выбранных лабораторий получили проходные баллы 90,0% или выше при проведении квалификационных серологических и молекулярных тестов на корь и краснуху. С 2018 года лаборатории входят в сеть лабораторий Индии для исследования кори и краснухи. В течение 12 месяцев после начала независимой отчетности шесть лабораторий протестировали 2287 образцов сыворотки и 701 мазок из горла или носоглотки или образец мочи.

**Вывод:**

Данный процесс привел к укреплению и расширению сети. Эта высокопрофессиональная лабораторная сеть помогла Индии расширить масштабы проведения серологических и молекулярных тестов на корь и краснуху, обеспечив при этом высокое качество тестирования. Модель сотрудничества, разработанная Правительством Индии и ВОЗ, может быть реализована другими странами в целях расширения сетей лабораторий для эпиднадзора за корью и краснухой, а также за другими инфекционными заболеваниями.

## Introduction

Countries in the World Health Organization (WHO) South-East Asia Region have extended their targets to eliminate the vaccine-preventable infections, measles and rubella, by 2023.^1^ These infections lead to substantial childhood morbidity and mortality.^2^ Measles mortality is particularly high in malnourished children residing in areas with poor health-care systems^2^^,^^3^ and rubella infection contracted during pregnancy can lead to miscarriage and congenital rubella syndrome.^4^^,^^5^

India is facing multiple challenges to meet these elimination targets. In 2019, the Indian government reported 10 430 measles cases and 3404 rubella cases to WHO.^6^ Active field surveillance along with a strong laboratory network is pivotal to the success of disease elimination programmes.^7^^–^^10^ In view of this requirement, field surveillance in India was strengthened and expanded, switching from outbreak to case-based surveillance and by 2019 all states had completed the transition. In the new surveillance approach, health workers collect serum and throat or nasopharyngeal swab or urine samples from individuals with suspected measles for laboratory confirmation and virus genotyping.^11^

To meet the increased testing demand due to the new surveillance approach, the Indian government made focused efforts to broaden the scope of several of the virus research and diagnostic laboratories in the country. This expansion would also strengthen and expand the national measles and rubella network, allow for a wider geographical coverage, improvements in turn-around time for testing and reduced shipment costs.

The WHO-coordinated global measles and rubella laboratory network was established to provide high-quality, standardized measles and rubella surveillance in six WHO regions in 2000.^12^ Being a part of the South-East Asia Region, the Indian measles and rubella laboratory network becomes an integral part of the WHO global laboratory network. To ensure testing uniformity across the network, laboratories need to follow the network’s testing requirements.^8^^–^^10^ An on-site annual accreditation review is conducted for each network laboratory to recognize them as being proficient.

In 2017, the Indian Council of Medical Research and WHO partnered to expand the measles and rubella laboratory network by integrating some of the virus research and diagnostic laboratories of the Indian Council of Medical Research and Department of Health Research, Government of India. Here, we describe the systematic 10-step scheme undertaken to integrate these laboratories into the measles and rubella laboratory network. We also describe challenges faced and lessons learnt during the expansion.

## Methods

The Institutional Ethical Committee of the Indian Council of Medical Research–National Institute of Virology approved the study (NIV/IEC/2018/November/D-38).

### Study setting

The measles and rubella laboratory network of India, initiated in 2003,^13^^,^^14^ consisted of 11 national and two reference laboratories in 2017. The King Institute of Preventive Medicine, Chennai, hosts the reference laboratory for serology and the Indian Council of Medical Research–National Institute of Virology, Mumbai Unit hosts the reference laboratory for molecular testing and sequencing. In 2019, the laboratories processed 18 000 serological tests and 5000 molecular tests (WHO India, personal communication, 27 January 2020).

The Indian government collaborates with WHO National Public Health Support Programme,^15^ which is involved in various vaccine-preventable disease surveillance activities, such as polio, measles and rubella. Field units in the programme collect samples from suspected measles and rubella cases, and these are sent to a laboratory in the network. This testing follows the WHO-India recommended test algorithm.^16^

In India, there were 106 virus research and diagnostic laboratories in 2019 with capability for serological and molecular testing of 20–25 medically important viruses. These laboratories are supported by the government for infrastructure, equipment, human resources and consumables for diagnosis. A small number of these laboratories can also perform genomic sequencing.^17^

### Selection of laboratories

To expand the national measles and rubella network, in 2017 the Indian Council of Medical Research and WHO selected six virus research and diagnostic laboratories based on the following criteria: (i) areas having high caseload but no measles and rubella laboratories, fully operational laboratory with good track record; (ii) proven capacity for serological and molecular diagnosis of any virus; (iii) good turn-around time for testing (≤ 72 hours); (iv) presence of trained human resources; (v) willingness to participate; (vi) good connectivity by air, rail and road; (vii) participation of these laboratories in other national programmes; and (viii) availability of computer and internet connection. The laboratories were further evaluated for satisfactory compliance with the external quality assurance programmes for arboviruses (dengue, chikungunya, Japanese encephalitis) and human influenza viruses, presence of operational equipment and annual maintenance contracts of equipment, having detailed standard operating procedures for diagnosis of different viruses in place and understanding of corrective preventive actions. 

### Obtaining WHO proficient status

Selected laboratories went through a 10-step scheme (Box 1), developed by the WHO country office for India and Indian Council of Medical Research. This scheme was developed to ensure compliance with WHO recommended protocols and quality assurance requirements.^16^ The scheme commenced with assessment of selected laboratories for enrolment in the network and ended with attainment of WHO proficient status by the successful laboratories.

Box 1 **10-step scheme for inclusion of a new laboratory in WHO measles and rubella laboratory network, 2017–2018**

Step 1: Pre-assessment visit to identify the strengths, weaknesses and areas for improvement.

Step 2: System strengthening in terms of ensuring personnel’s willingness to participate, improving infrastructure and buying equipment.

Step 3: Hands-on training on recommended methods.

Step 4: Testing of practice samples to gain proficiency in test experiments.

Step 5: Testing of unknown samples: should achieve more than 90.0% concordance.

Step 6: Parallel testing of surveillance samples and validation of first 50 samples (sera) by King Institute of Preventive Medicine, Chennai for serology: should achieve 90.0% or more concordance. Training on data management using Measles Laboratory Information System.

Step 7: Independent testing and reporting to national measles and rubella surveillance programme.

Step 8: Testing of proficiency test panel as part of external quality assurance.

Step 9: On-site visit by expert: laboratory gaining status of proficient laboratory should achieve 80.0% or more on the accreditation review.

Step 10: Laboratory will gain status of proficient laboratory.

#### Step 1

To evaluate the laboratories’ readiness to undertake additional work of increased measles and rubella testing, the Indian Council of Medical Research undertook an on-site pre-assessment of each laboratory. This assessment included evaluation of available infrastructure, equipment, human resources, existing laboratory quality management systems, willing responsible officer and areas where strengthening and improvement was required.

#### Step 2

Laboratories ensured personnel’s willingness to participate in the network, improved infrastructure and bought equipment if needed, by using Indian government funds received when they integrated under the virus research and diagnostic laboratories scheme. 

#### Step 3

At the National Institute of Virology, Mumbai Unit, WHO and the reference laboratories provided a 5-day practical training to the laboratories. Training covered how to perform the enzyme-linked immunosorbent assay (ELISA) and the conventional reverse transcription polymerase chain reaction (PCR) method as per the WHO recommended protocols.^16^ WHO also invited trainers from the National Institute of Health, Thailand, for this training. The Indian Council of Medical Research facilitated the training by providing funds, facility, facilitators and all logistics support for in-country participants.

#### Step 4

The Indian Council of Medical Research–National Institute of Virology, Mumbai Unit provided the laboratories with practice panels. The serology practice panel comprised of 10 samples containing 400 µL serum each of measles and rubella IgM positive and negative sera. These were tested both for measles and rubella IgM. Sera for practice panels had been received through the surveillance network and tested at the National Institute of Virology, Mumbai Unit. We selected sera with optical density values of 0.4–1.0 and of good quality (no haemolysis, non-lipolytic). WHO country office for India provided the reagents: Enzygnost® Anti-Measles-Virus/immunoglobulin (IgM) and Enzygnost® Anti-Rubella-Virus/IgM ELISA kits (Siemens Diagnostics Products, Marburg, Germany). Laboratories were requested to submit their reports to the Indian Council of Medical Research–National Institute of Virology, Mumbai Unit, within 14 days of receipt of samples for review and scoring.

Molecular practice panels comprised of viruses spotted on Whatman® Flinders Technology Associates® cards (Cytiva, Tokyo, Japan), provided by the WHO country office.^18^ Each panel consisted of two positive and three negative measles samples and two positive and three negative rubella samples. The National Institute of Virology prepared the panel by spotting 200 µL virus isolates from the institute’s repository on each positive card. The negative cards were spotted with Dulbecco’s Minimal Essential Media (Cat no. 09123354, Himedia, Mumbai, India). Spotted cards were dried and packed in zip lock bags containing desiccant pouches and distributed to the laboratories at room temperature. The molecular reference laboratory provided primers and reagents. Primers were received from United States Centers for Disease Control and Prevention (CDC) through the International Reagent Resource (CDC, Atlanta, United States of America, USA) and the molecular reference laboratory locally procured the reagents. The laboratories tested these panels using the WHO recommended methods,^16^ as instructed during the training. Laboratories sent their positive PCR products for sequencing to the molecular reference laboratory and subsequently received the sequences for further analysis. Laboratories were requested to submit their reports to the Indian Council of Medical Research–National Institute of Virology, Mumbai Unit, within 30 days of receipt of panel for review and scoring.

#### Step 5

After completion of practice panels, the two reference laboratories sent the laboratories unknown samples for testing. The unknown panel comprised of 40 serology and 10 molecular testing samples, prepared as mentioned above. Laboratories achieving a passing score of 90.0% or more (concordance of ≥ 36/40 samples in serology panel and ≥ 9/10 samples in molecular panel) were then allowed to receive samples directly from the WHO National Public Health Support Programme.^15^

#### Step 6

To validate the laboratories, the serology reference laboratory performed parallel testing by instructing the laboratories to make two aliquots of the first 50 serum samples received from the WHO National Public Health Support Programme. If the sample number was lower than 50, 10.0% positive and 10.0% negative of the total samples was used. One aliquot was for their own testing and the other was to be referred to the serology reference laboratory. 

Simultaneously the WHO National Public Health Support Programme trained the laboratories on data management on the WHO India measles laboratory information system software.

#### Step 7

To be allowed to report their results independently to the national surveillance programme, the laboratories needed to achieve concordance of 90.0% or more of the panel results (as per the WHO accreditation checklist; available in the data repository)^19^ in the parallel testing.

In April 2019, WHO and the Indian Council of Medical Research with CDC organized a training on sequence analysis using GeneStudio software (Informer technologies Inc., Los Angeles, USA) and submission of sequences in WHO measles and rubella nucleotide surveillance MeaNS and RubeNS database.^20^^–^^23^

#### Step 8

The laboratories enrolled in an external quality assurance scheme and participated in a global proficiency test. All panels were procured by WHO. The Victorian Infectious Diseases Reference Laboratory, Melbourne, Australia provided serology panels, which comprised of 20 measles and rubella IgM positive and negative sera. Results were reported to the Victorian Infectious Diseases Reference Laboratory website within 14 days of receipt of the panel. The Viral Vaccine Preventable Diseases Branch, CDC, Atlanta, USA, provided five spotted cards as a molecular panel. Laboratories submitted the results in CDC’s reporting format within six weeks of panel receipt. The laboratories submitted the sequences to MeaNS/RubeNS proficiency test panel portal for review.

#### Step 9

International experts supported by WHO assessed laboratories on-site as per the WHO measles and rubella accreditation checklist (available in the data repository).^19^ During the assessment, experts comprehensively evaluated: laboratory layout; implementation of good laboratory practices; laboratory techniques, staff training and staff vaccination records; biosafety and biosecurity aspects; laboratory quality management systems; and documentation, including logbooks, equipment calibration and standard operating procedures. The experts provided recommendations for further improvement, such as biosafety and good laboratory practices, documentation and regular supervision of activities involved.

#### Step 10

In the final step, WHO designated laboratories with a ≥ 80.0% score on the WHO accreditation checklist as a WHO proficient laboratory and included them in the national measles and rubella network.

## Results

During the first phase of expansion, the Indian Council of Medical Research and WHO selected six laboratories for obtaining proficiency status and inclusion in the network (Table 1). 

**Table 1:** Measles and rubella diagnosis by the newly integrated laboratories in the Indian measles and rubella laboratory network, 2018–2019

Laboratory	Location	Type of test								Sera cross-verification score, % (no. of test results correct/no. of tests)^c^	
		Serology				Molecular					
		No. of samples tested^a^	No. (%) of positive tests			No. of samples tested^b^	No. (%) of positive tests			Measles	Rubella
			Measles	Rubella			Measles	Rubella			
Indian Council of Medical Research–National Institute of Virology, Kerala Unit	Alappuzha	786	403 (51.3)	6 (0.8)		185	101 (54.6)	0 (0.0)		98.0 (49/50)	100.0 (28/28)
Rajendra Institute of Medical Science	Ranchi	230	25 (10.9)	13 (5.7)		55	3 (5.5)	0 (0.0)		92.0 (46/50)	100.0 (41/41)
Indian Council of Medical Research–Regional Medical Research Centre	Bhubaneswar	930	149 (16.0)	73 (7.8)		103	0 (0.0)	0 (0.0)		94.0 (47/50)	100.0 (34/34)
Indian Council of Medical Research–Regional Medical Research Centre	Port Blair	10^d^	1 (10.0)	0 (0.0)		10	0 (0.0)	0 (0.0)		100.0 (8/8)	100.0 (8/8)
Indian Council of Medical Research–National Institute for Research in Tribal Health	Jabalpur	287	42 (14.6)	23 (8.0)		316	49 (15.5)	3 (0.9)		100.0 (25/25)	100.0 (16/16)
Hassan Institute of Medical Sciences	Hassan	44	5 (11.4)	1 (2.3)		32	4 (12.5)	0 (0.0)		100.0 (21/21)	100.0 (21/21)

^a^ Serum sample.

^b^ Urine sample or throat swab.

^c^ The score is based on concordance of testing results between the newly integrated laboratory and reference laboratory. The score was calculated on 50 samples annually or 10.0% positive and 10.0% negative of the total samples tested for serology.

^d^ Due to geographical location the laboratory has very low workload.

During on-site assessment in 2017–2018, all six laboratories expressed willingness to accept additional workload of measles and rubella surveillance within existing human resources. A biosafety cabinet was provided to one laboratory as part of system strengthening.

**Fig. 1:**
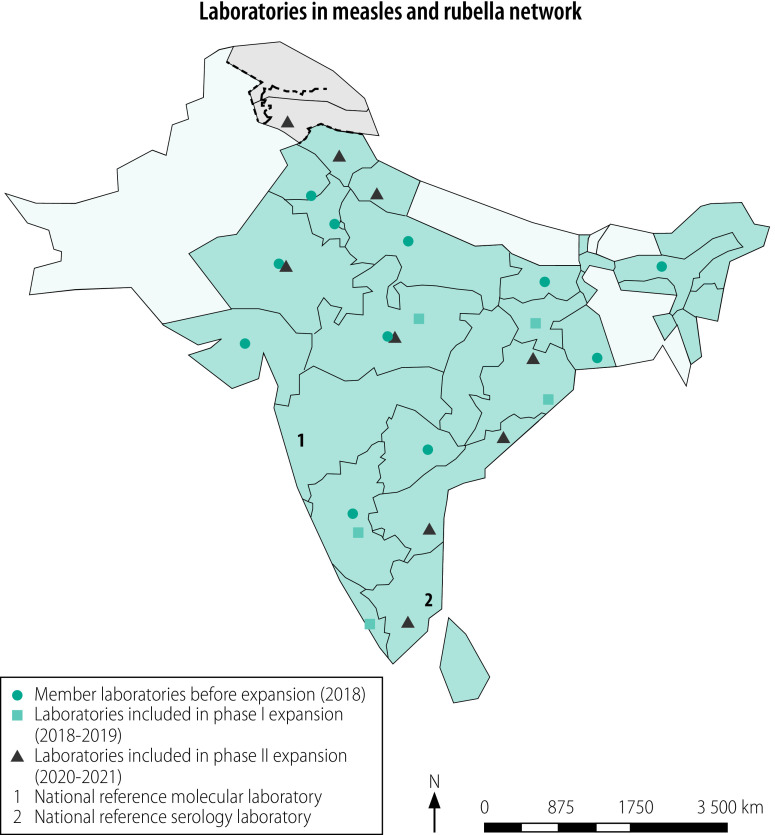
Serology testing performance of newly integrated laboratories in the Indian measles and rubella laboratory network, 2018–2019

Notes: The *x*-axis presents the location of the laboratories; the full name of these laboratories are given in Table 1. The serology test was an immunoglobulin M enzyme-linked immunosorbent assay. The practice panels consisted of 10 samples and the unknown panels 40 samples. Laboratories performed parallel testing of 50 serum samples with the reference laboratory, except the Indian Council of Medical Research–Regional Medical Research Centre laboratory in Port Blair that only tested eight serum samples due to low workload.

**Fig. 2:**
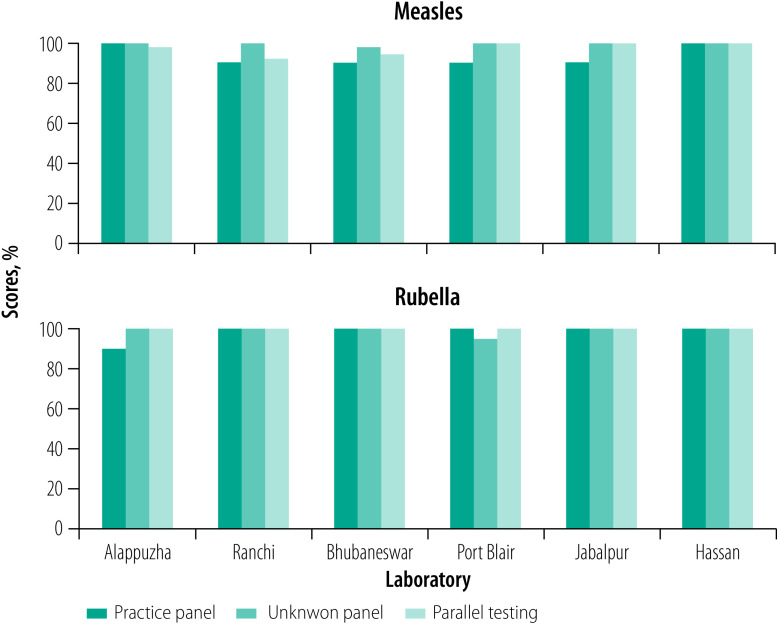
Molecular testing performance of newly integrated laboratories in the Indian measles and rubella laboratory network, 2018–2019

Notes: The *x*-axis presents location of the laboratories; the full name of these laboratories are given in Table 1. The molecular test was a conventional reverse transcription polymerase chain reaction. The practice panels consisted of five samples and the unknown panels 10 samples.

For the measles practice panel, two laboratories scored 100.0% (10/10) while four scored 90.0% (9/10). In the rubella practice panel, one laboratory scored 90.0% (9/10) while the rest scored 100.0% (10/10). The Indian Council of Medical Research–National Institute of Virology, Mumbai Unit carried out trouble shooting with laboratories to identify areas for improvement. The laboratory received information about important factors that can affect the final results of serology assay such as maintaining desired room temperature, pipetting techniques and incubation periods. As a result, five laboratories achieved a 100.0% (40/40) score in the unknown panel for measles and rubella serology, while one laboratory scored 97.5% (39/40) for measles and one laboratory 95.0% (38/40) for rubella (Fig. 1). In the molecular panel, all laboratories scored 100.0% (10/10) on the measles testing, while five laboratories achieved a 100.0% (10/10) score on the rubella testing and one laboratory 90.0% (9/10; Fig. 2).

After successful completion of step 5, the laboratories’ staff members attended data management training. Step 6 also covered parallel testing for which all six laboratories achieved a passing score of 90.0% or more in serology (Fig. 1). Subsequently, WHO country office for India instructed the field units of the WHO National Public Health Support Programme in catchment areas of the new laboratories to send samples directly to the laboratories for testing. Considering their overall performance, all laboratories initiated independent reporting to the national surveillance programme through the information system under Step 7. Simultaneously, the laboratories initiated submission of positive PCR products to the reference laboratory for sequencing. The training on analysing sequences of measles and rubella enabled the laboratories to identify genotype and independently submit data in the WHO MeaNS and RubeNS database. In Step 8, as part of the external quality assurance scheme, the laboratories participated for the first time in the WHO global proficiency test panel for both serology and molecular tests. All six laboratories successfully achieved passing scores of 90.0% or more in both the panels.

Four of the six laboratories also underwent on-site WHO accreditation review by international experts between September and October 2019. Two laboratories achieved passing scores of  90.0% or more, whereas the other two achieved more than 80.0% scores (further information available in authors’ data repository).^19^ Due to the unforeseen coronavirus disease 2019 (COVID-19) pandemic, the on-site assessment of the remaining two laboratories (one in Hassan and one in Port Blair) had to be postponed. However, these two laboratories submitted an internal audit report to the WHO National Public Health Support Programme for review and will undergo the WHO accreditation process once the pandemic situation improves. 

With the successful implementation and completion of the 10-step scheme, the six laboratories were integrated as subnational laboratories into the Indian measles and rubella laboratory network. The entire activity of inclusion of the laboratories in the network took approximately 24 months. Even after initiation of independent testing, these laboratories were closely monitored for their performance indicators, results of cross-verification for serology and timeliness of result reporting. Within 12 months of initiation of independent reporting, the six laboratories have tested a total of 2287 sera and 701 throat or nasopharyngeal swabs or urine samples (Table 1). In future, each laboratory is expected to process a minimum of 3000 serology and 750 molecular testing samples per year. However, these numbers may differ among laboratories.

**Fig. 3:**
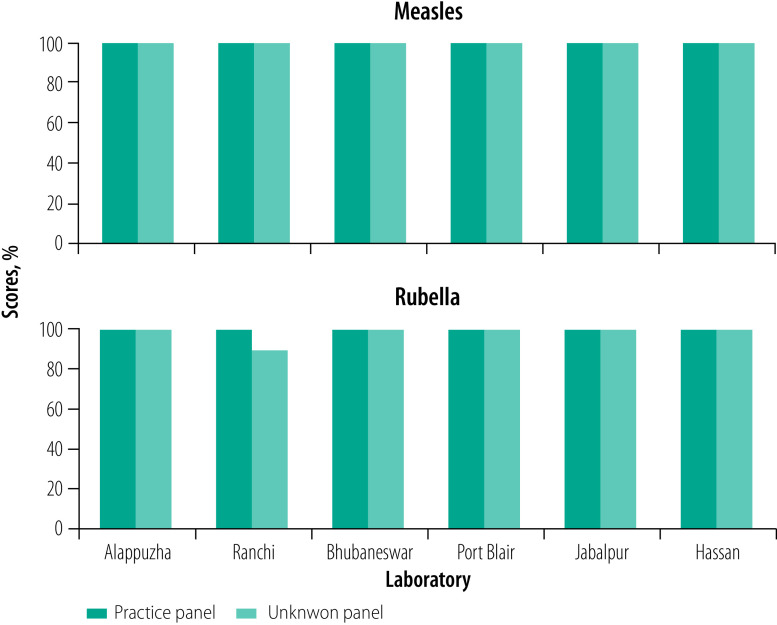
Timeliness of reporting of measles and rubella samples of newly integrated laboratories in the Indian measles and rubella laboratory network, 2018–2019

Notes: The *y*-axis presents location of the laboratories; the full name of these laboratories are given in Table 1. Timeliness of reporting serology results within four days of sample receipt and molecular results within 30 days.

During a 12-month period, all laboratories except one have maintained 90.0% or more reporting timeliness for serology (Fig. 3). Three out of the six laboratories were successful in maintaining the reporting timeliness of 90.0% or more for molecular testing. The remaining laboratories could not maintain the timeliness due to repurposing of human resources and consumables for COVID-19 diagnostic testing. 

For quality assurance the laboratories participated in cross-verification or confirmatory testing of sera with the serology reference laboratory in 2018–2019 (Table 1). During cross-verification, each laboratory had to send 50 samples annually or 10.0% positive and 10.0% negative of the total samples tested for serology. All six laboratories have achieved > 90.0% cross-verification scores for both measles and rubella, which has helped them in achieving WHO proficient status and integration into the global measles and rubella laboratory network. 

## Discussion

Regional elimination of measles and rubella to achieve eradication is one of the top global public health priorities.^3^ To meet targets for elimination, WHO has adopted a strategic plan for elimination of measles and rubella, containing five objectives: (i) achieving and maintaining high population immunity with ≥ 95.0% two dose measles and rubella vaccination coverage in all parts of each country; (ii) developing and sustaining a sensitive and timely case-based surveillance; (iii) developing and maintaining a proficient laboratory network; (iv) ensuring adequate outbreak preparedness and response; and (v) strengthening support and linkages.^24^^,^^25^ While the WHO National Public Health Support Programme, India along with the Indian government are addressing all of the objectives, here we only describe how they have addressed objective (iii) in the strategic plan. To do so, six virus research and diagnostic laboratories were engaged in a 10-step scheme developed by WHO and the Indian Council of Medical Research. This process aimed to ensure seamless integration to the national measles and rubella laboratory network while initiating high quality independent testing through laboratories obtaining WHO proficiency status.

Implementation of the scheme was challenging. Selecting appropriate laboratories within the required catchment areas, fulfilling the requirements of the inclusion checklist, having willing responsible officers, engaging international trainers, developing practice and external quality assurance panels were burdensome. However, the biggest challenge was to re-orient the laboratories to align the laboratory systems, quality assurance and controls requirements and data entry as per WHO global network requirements. Before the laboratories’ inclusion in the network, they were already testing for measles and rubella in samples referred to them from the Integrated Disease Surveillance Programme, which is not a part of the WHO global network mechanism. They therefore had a completely different channel of data entry and quality assurance and quality controls before joining the network. Realigning them to a different mechanism for the WHO global network proved to be difficult but was achieved through rigorous follow-ups and trainings. Also, shortage of serology kits due to discontinuation of Siemens kits because of unforeseen problems with the manufacturer mandated the need to standardize testing with Euroimmun kits (Euroimmun AG, Lübeck, Germany). This shortage delayed the timelines of operationalization of the new laboratories. Meeting the sudden increase in demand for molecular testing reagents was also challenging for the laboratories. However, coordinated efforts by the Indian Council of Medical Research and the Ministry of Health and Family Welfare helped in overcoming the crisis by supporting the provision of serology diagnostic kits, while WHO provided molecular diagnostic reagents. The Indian Council of Medical Research and The Ministry of Health and Family Welfare also earmarked separate budgets for repeated trainings, on-site visits and shipment of clinical specimens. Existing resources at the laboratories in terms of human resources, equipment and infrastructure were used for undertaking the extra workload of the measles and rubella network. The Indian Council of Medical Research and WHO incurred additional costs, such as trainings and travel, panel preparation, diagnostic and consumables procurement and shipment. The approximate cost incurred on these requirements was 0.23 million United States dollars.

Overall, the 10-step scheme had several advantages in addition to laboratories obtaining WHO proficient status. The process helped in raising awareness of laboratories towards the importance of strict adherence to test protocols along with good laboratory practices, highlighting the weaknesses of some laboratories and pointing out the areas requiring improvement and strengthening, instilling confidence in laboratories which repeatedly achieved passing scores. This exercise also helped the laboratories in overall improvement of quality in all other testing areas, such as arboviruses, respiratory viruses and enteric viruses.

Following the successful integration, the Indian Council of Medical Research and WHO have identified nine more virus research and diagnostic laboratories. These laboratories are in the advanced stages of integration into the national network by following the 10-step scheme. 

Successful measles and rubella laboratory strengthening in India has helped in improving the turn-around time of testing and guiding field surveillance teams to take appropriate public health interventions for case management and curtailing transmission, thus aligning India’s efforts towards regional measles and rubella elimination (Ministry of Health and Family Welfare, Government of India, unpublished data, 31 December 2021). The expansion also supported the transition to a more sensitive method of measles surveillance. After a successful pilot in 2018 in three states, where the measles case definition no longer included the symptoms coryza, conjunctivitis and cough, and instead used fever and rash surveillance, the entire country transitioned to such a method of surveillance in 2021. In the future, the expanded network will also help in meeting the key surveillance performance indicators of at least two non-measles, non-rubella discarded fever and rash cases per 100 000 population at national level.^26^^,^^27^

The 10-step scheme presented here can serve as a guiding tool for countries planning to enhance national laboratory capacity, following global standards. While the scheme can be deployed for measles and rubella laboratory expansion, it can also be used for strengthening and expanding laboratory networks for surveillance and early detection of pathogens of public health importance.
